# A novel *Gardnerella*, *Prevotella*, and *Lactobacillus* standard that improves accuracy in quantifying bacterial burden in vaginal microbial communities

**DOI:** 10.3389/fcimb.2023.1198113

**Published:** 2023-06-19

**Authors:** Jacob H. Elnaggar, Caleb M. Ardizzone, Nuno Cerca, Evelyn Toh, Paweł Łaniewski, Rebecca A. Lillis, Melissa M. Herbst-Kralovetz, Alison J. Quayle, Christina A. Muzny, Christopher M. Taylor

**Affiliations:** ^1^ Department of Microbiology, Immunology, and Parasitology, Louisiana State University Health Sciences Center, New Orleans, LA, United States; ^2^ Laboratory of Research in Biofilms Rosário Oliveira (LIBRO), Centre of Biological Engineering (CEB), University of Minho, Braga, Portugal; ^3^ Department of Microbiology & Immunology, Indiana University School of Medicine, Indianapolis, IN, United States; ^4^ Department of Basic Medical Sciences, College of Medicine-Phoenix, University of Arizona, Phoenix, AZ, United States; ^5^ Division of Infectious Diseases, Department of Medicine, Louisiana State University Health Sciences Center, New Orleans, LA, United States; ^6^ Division of Infectious Diseases, University of Alabama at Birmingham, Birmingham, AL, United States

**Keywords:** bacterial burden, vaginal microbiome, bacterial vaginosis, biofilm, qPCR standard, *Gardnerella*, *Prevotella*, *Lactobacillus*

## Abstract

Bacterial vaginosis (BV) is the most common vaginal dysbiosis. In this condition, a polymicrobial biofilm develops on vaginal epithelial cells. Accurately quantifying the bacterial burden of the BV biofilm is necessary to further our understanding of BV pathogenesis. Historically, the standard for calculating total bacterial burden of the BV biofilm has been based on quantifying *Escherichia coli* 16S rRNA gene copy number. However, *E. coli* is improper for measuring the bacterial burden of this unique micro-environment. Here, we propose a novel qPCR standard to quantify bacterial burden in vaginal microbial communities, from an optimal state to a mature BV biofilm. These standards consist of different combinations of vaginal bacteria including three common BV-associated bacteria (BVAB) *Gardnerella* spp. (G), *Prevotella* spp. (P), and *Fannyhessea* spp. (F) and commensal *Lactobacillus* spp. (L) using the 16S rRNA gene (G:P:F:L, G:P:F, G:P:L and 1G:9L). We compared these standards to the traditional *E. coli* (E) reference standard using known quantities of mock vaginal communities and 16 vaginal samples from women. The E standard significantly underestimated the copy numbers of the mock communities, and this underestimation was significantly greater at lower copy numbers of these communities. The G:P:L standard was the most accurate across all mock communities and when compared to other mixed vaginal standards. Mixed vaginal standards were further validated with vaginal samples. This new G:P:L standard can be used in BV pathogenesis research to enhance reproducibility and reliability in quantitative measurements of BVAB, spanning from the optimal to non-optimal (including BV) vaginal microbiota.

## 1 Introduction

Bacterial vaginosis (BV) is the most common vaginal infection, affecting more than 30% of women in the United States (Bautista et al., 2016). Although the exact etiology is yet to be identified, BV is known to be associated with loss of protective lactic acid- and hydrogen peroxide-producing *Lactobacillus* spp. and a dramatic increase in facultative and strict anaerobic bacteria (BV-associated bacteria; BVAB) (from 10^7^ to 10^9^ bacterial genomes per sample) including *Gardnerella* spp. (10^7^)*, Prevotella* spp. (10^8^), and *Fannyhessea vaginae* (10^6^) (Zozaya-Hinchliffe et al., 2010; Ravel et al., 2011). The current recommended treatment regimens for BV consist of oral and intra-vaginal metronidazole and clindamycin (“Bacterial Vaginosis - 2021 Sexually Transmitted Diseases Treatment Guidelines”, 2021), each with high initial success rates (80%). However, more than 60% of women will have an episode of recurrent BV (Bradshaw et al., 2006).

BV is associated with the formation of a polymicrobial biofilm on the surface of vaginal epithelial cells, which likely contributes to high recurrence rates after treatment (Patterson et al., 2007; Swidsinski et al., 2008). The sequence of events leading to BV biofilm formation is controversial and under active study (Machado et al., 2016; Sousa et al., 2023). One hypothesis is that a virulent strain of *G. vaginalis*, likely sexually transmitted, is the primary pathogen that displaces protective *Lactobacillus* spp. and adheres to the vaginal epithelium, initiating BV biofilm formation and allowing secondary colonizers to attach and multiply (Patterson et al., 2010; Santiago et al., 2011). In this hypothetical model, one additional early colonizer is thought to be *P. bivia*, which is recruited into the lower layers of the biofilm that is initiated by *G. vaginalis* (Verstraelen et al., 2009). Vaginal sialidase, produced by both *G. vaginalis* and *P. bivia*, promotes breakdown of the protective mucous layer on the vaginal epithelium (Wiggins et al., 2001). Loss of the protective mucous layer on the vaginal epithelium leads to increased adherence of other BVAB, including *F. vaginae* (Hardy et al., 2015), which join the BV biofilm in the upper layers, leading to the formation of a mature, polymicrobial entity (Castro et al., 2021). A better understanding of the development of the polymicrobial BV biofilm is crucial for improving BV diagnosis and developing more effective treatments.

Determining the burden of specific BVAB is important for better understanding the pathogenesis of incident BV (Gajer et al., 2012; Muzny et al., 2018). The 16S ribosomal ribonucleic acid (rRNA) gene, present in all bacterial genomes, is used to estimate bacterial burden (Woese et al., 1990). Polymerase chain reaction (PCR) amplification and sequencing of hypervariable regions of the 16S rRNA gene has been widely applied to characterize the vaginal microbiota and further investigate BV pathogenesis. However, this method only provides the relative abundance of each micro-organism, rather than absolute quantities (Poretsky et al., 2014). Quantitative PCR (qPCR) targeting the 16S rRNA gene is commonly used to measure the burden of bacterial species (Santiago et al., 2012; Ricchi et al., 2017; Galazzo et al., 2020). Typically, these assays use plasmid standards containing the *Escherichia coli* 16S rRNA gene. The *E. coli* (E) reference standard is a common tool for measuring bacterial burden in many types of communities (Zozaya-Hinchliffe et al., 2010; Jian et al., 2020; Tettamanti Boshier et al., 2020), however, this approach has not been evaluated for its accuracy in vaginal samples. Generally, universal primers amplify 16S rRNA genes from different genera with varying efficiencies due to differences in intervening sequences (e.g., guanine or cytosine [GC] content and length) (Aird et al., 2011), and *E. coli* is not a natural colonizer of the vaginal micro-environment (Acinas et al., 2004; Větrovský and Baldrian, 2013). Therefore, the use of an E standard may influence the results of the quantification of 16S rRNA gene copy number within the vaginal microbiota.

Accurate quantitation of bacterial burden in complex vaginal communities requires specifically designed standards. We generated standards specific for common vaginal micro-organisms to establish a method that allows for estimation of the bacterial burden of the vaginal micro-environment, from an optimal state to a mature, polymicrobial BV biofilm. These novel standards are composed of *Gardnerella* spp. (G)*, Prevotella* spp. (P)*, Fannyhessea* spp. (F), and *Lactobacillus* spp. (L) were compared to the traditional E standard using mock vaginal communities and patient-derived vaginal samples.

## 2 Materials and methods

### 2.1 Vaginal samples

We used stored isolated deoxyribonucleic acid (DNA) from vaginal swabs that were previously obtained in a study described in Mott et al., 2021 (Louisiana State University Health Sciences Center [LSUHSC] IRB protocol #1081). In brief, vaginal samples were collected from women seeking care at the LSUHSC CrescentCare Sexual Health Center Clinic in New Orleans, LA using a Copan Swab (Copan, Murrieta, CA, USA). DNA extraction and sequencing were performed by the LSUHSC Microbial Genomics Resource Group. Genomic DNA was extracted using the QIAamp DNA Stool Mini Kit (Qiagen, Hilden, Germany), modified to include bead beating. In addition to a swab being used for sequencing, a swab was also used for Gram stain and Nugent scoring to characterize the vaginal microbiome (Nugent et al., 1991). BV was common in this population of women (38% by Nugent score) and representative samples were selected from across the spectrum of the vaginal microbiota, characterized by Nugent score (n=6 normal vaginal microbiota, 5 intermediate vaginal microbiota, and 5 BV) and ethnicity (n=8 black, 7 white, and 1 other). Complete metadata is listed in **Table S1B**. Bacterial relative abundance can be observed in heatmaps (**Figure S2**) (Agile Toolkit for Incisive Microbial Analysis).

### 2.2 Primer design

**Figure 1:**
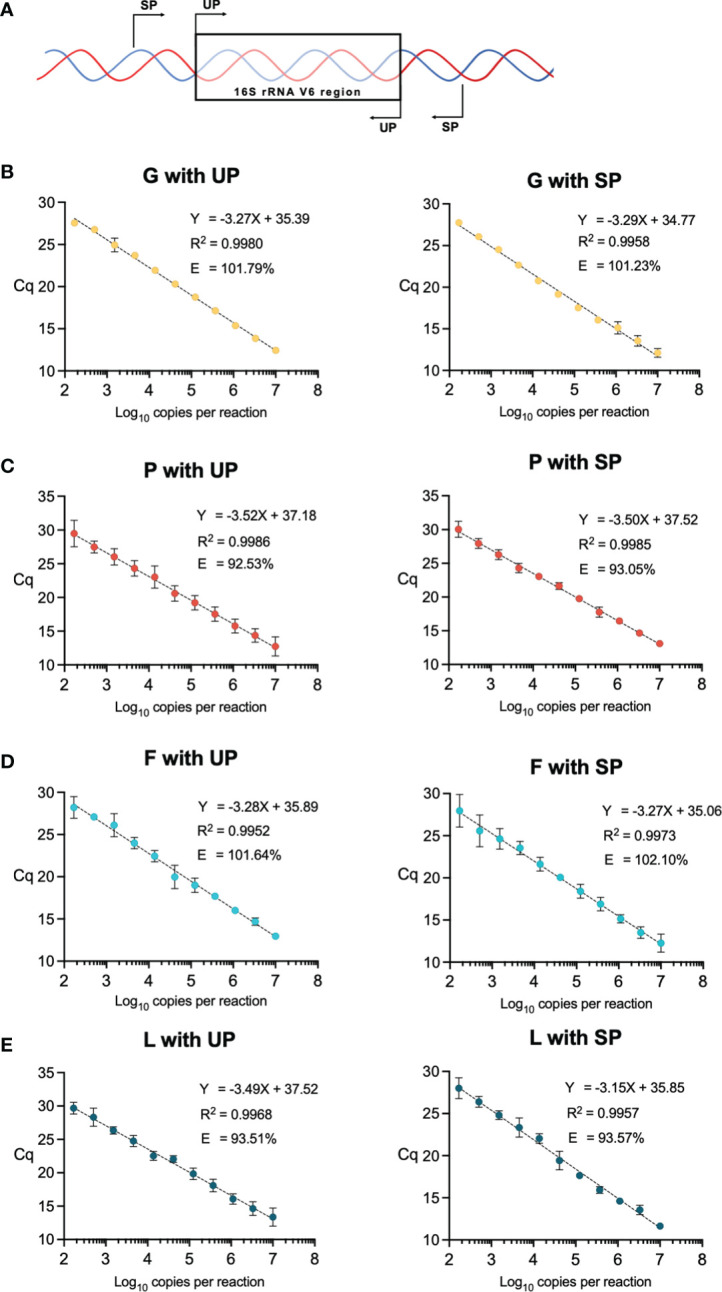
**(A)** Diagram of universal primer (UP) and specific primer (SP) locations in the V6 region of the 16S rRNA gene. **(B–E)** qPCR standards using the SP and UP for each vaginal bacteria of interest, *Gardnerella* spp. **(G)**, *Prevotella* spp. (P), and *Fannyhessea* spp. **(F)**, and *Lactobacillus* spp. (L). Data points are generated from three-fold dilutions of the purified plasmids starting at 10^7^ copies to 167 copies per reaction. Each point represents an average of 3 replicate qPCR reactions with corresponding error bars. Efficiency **(E)** is calculated based on the slope of the linear regression. Axes are labeled by the quantification cycle (Cq) and the number of copies per reaction (log10).

We used published universal qPCR primers (UP) targeting the sixth hypervariable region (V6) of the bacterial 16S rRNA gene of multiple bacterial taxa (Bacchetti De Gregoris et al., 2011). We also designed taxa-specific primers (SP) outside the V6 amplicon of the UP for our bacterial taxa of interest, *Gardnerella* spp., *Prevotella* spp., *Fannyhessea* spp.*, Lactobacillus* spp., and *E. coli* (**Figure 1A**). We developed python and bash programs to computationally nominate specific primers with set parameters (GC content: >50%, melting temperature: 55-65°C, and primer length: 18-22 bp) and validated them using BLAST queries (https://github.com/elnaggarj/16S-primer-design) (Altschul et al., 1990). Once chosen, these primers were synthesized (Integrated DNA Technologies, Coralville, IA) and sensitivity and specificity was tested via PCR using vaginal samples and American Type Culture Collection (ATCC) bacterial isolates when available; *G. vaginalis* ATCC 10287, *L. crispatus* ATCC 33197, and *E. coli* ATCC 25922.

### 2.3 qPCR conditions

Each qPCR reaction contained 0.5 nmol of primers, 1 µl of template, and 7 µl of SsoAdvanced Universal SYBR Green Supermix (Bio-Rad, Hercules, CA) in a total of 10 µl. The qPCR conditions included a premelt at 98°C for 3 minutes, and then 40 cycles of 98°C for 15 seconds and 60°C for 15 seconds, followed by a final melt curve where the temperature was incrementally increased 0.5°C for 5 seconds from 65°C to 98°C (**Figure S1A**). All qPCR reactions were performed in triplicate and values are listed in **Table S1A**.

### 2.4 Standard curve generation

Standard curves were generated for bacteria taxa of interest, *Gardnerella* spp., *Prevotella* spp., *Lactobacillus* spp., *Fannyhessea* spp., and *E. coli*. The rRNA gene was amplified for each bacterial micro-organism of interest from vaginal samples, and the *E. coli* rRNA gene was amplified from the ATCC strain using the SP. Specific V6 amplicons for each bacterial micro-organism was generated using OneTaq 2X Master Mix with Standard Buffer (New England Biolabs [NEB] Ipswich, MA). 16S rRNA gene amplicons for each micro-organism were sequenced and verified to be identical to the reference genome (Eurofins genomics, Louisville, KY, USA). For *Gardnerella* spp. we compared the amplicon to the *G. vaginalis* ATCC 10287, *Prevotella* spp. to *P. bivia* ATCC 29303, *Fannyhessea* spp. to *F. vaginae* ATCC BAA-55, *Lactobacillus* spp. to *L. crispatus* ATCC 33197, and *E. coli* to *E. coli* ATCC 25922. These amplicons were ligated into a pGEM-T Easy vector (Promega, Madison, WI) (**Figure S1B**) and transformed into chemically competent DH5α *E. coli* (Invitrogen Waltham, MA) to isolate single colonies (Bertani, 2004). We performed validation for correct inserts via PvuII-HF (NEB) digests on gel and Sanger sequencing (Eurofins, Luxembourg). Vectors/plasmids with specific amplicons for each vaginal bacterial organism were purified using HiSpeed Plasmid Maxi Kit (Qiagen). Concentrations of purified vectors were measured using a NanoDrop 2000 (Thermo Scientific, Waltham, MA). The copy number of each vector was calculated from concentrations derived from quantitative gel densitometry. qPCR standards for each bacterial taxa of interest were generated from ten three-fold dilutions of the purified plasmids starting at 10^7^ copies to 10^2^ copies per reaction. Taxa-specific standards were then amplified using SP and UP. Vaginal mix standards and E standard assays were amplified with UP (Bacchetti De Gregoris et al., 2011). The regression coefficients and reaction efficiencies, based on the slope of the regression (-1 + 10^-1/slope^), were compared for each standard. A 100% efficiency indicated that the PCR product of interest was accurately doubling with each cycle (Stolovitzky and Cecchi, 1996), and an appropriate efficiency is between 90-110%.

### 2.5 Mock vaginal communities

*Gardnerella* spp., *Prevotella* spp., *Fannyhessea* spp., and *Lactobacillus* spp. 16S rRNA genes in plasmids were combined in known quantities to simulate mock vaginal communities. Mock vaginal communities were generated at 10^6^, 10^5^, 10^4^ total copy numbers, and quantified using both the E standard and the mixed vaginal standards. The calculated output from each standard was compared to the known input of each mock vaginal community. The output was divided by the input to generate a percentage. A 100% output/input indicated that identical input and output copy numbers were read from the standard.

### 2.6 Assessing standards using vaginal samples

Three ten-fold dilutions were made from the original isolated DNA from the vaginal samples. The 16S rRNA gene copy number per reaction was measured using the E and mixed vaginal standards to determine if there was a change in the difference between the two standards at different dilutions. Next, 16S rRNA gene copy number per sample was calculated and compared between the two standards and across Nugent categories (normal vaginal microbiota [0-3], intermediate vaginal microbiota [4-6], and BV [7-10]) (Nugent et al., 1991).

### 2.7 Statistical analysis and power

Analyses were performed using Prism (version 9.3.1; GraphPad Software, Inc., La Jolla, CA). Linear regressions were performed on standard curves. qPCR-derived quantification cycle (Cq) value was converted to 16S rRNA gene copies and compared using two-way analysis of variance (ANOVA). Test statistics and p-values are listed in **Table S1C**. Comparing 16 vaginal samples, we have over 95% power to detect differences between the E and vaginal standard using a two-tailed Type I error rate of 0.05.

## 3 Results

### 3.1 Taxa-specific primers to 16S rRNA gene

SP were generated for: *Gardnerella* spp. (G), *Prevotella* spp. (P), *Fannyhessea* spp. (F), *Lactobacillus* spp. (L), and *E. coli* (EC) (primer sequences listed in **Table S1A**), and quantitative standards for each species using SP and UP were generated from three-fold dilutions of the purified plasmids from 10^7^ copies to 10^2^ copies per reaction (**Figures 1B–E**). The length of the UP amplicon was similar across all tested bacterial species and the SP amplicon varied between 230 and 290 base pairs (**Table 1**). Although there was a difference in length between the UP and SP for a given micro-organism, the efficiency did not vary more than 1% (**Table 1**). Furthermore, there was no notable difference in the R^2^ of the standards and all R^2^ were greater than 0.99, meaning that the standards approximated a linear slope.

**Table 1:** Length and efficiencies of standards.

Standard	UP amplicon size (bp)	SP amplicon size (bp)	UP Efficiency (%)	SP Efficiency (%)
*Gardnerella* spp. (G)	175	290	101.79	101.23
*Prevotella* spp. (P)	175	290	92.53	93.05
*Fannyhessea* spp. (F)	174	230	101.64	102.10
*Lactobacillus* spp. (L)	174	253	93.51	93.57
*E. coli* (E)	174	275	96.10	NA
G:P:F:L	NA	NA	96.49	NA
G:P:F	NA	NA	92.38	NA
G:P:L	NA	NA	95.30	NA
1G:9L	NA	NA	95.25	NA

UP, universal primers; SP, specific primers; bp, base pairs.

NA, not applicable.

### 3.2 Vaginal specific standard for bacterial burden

As the taxa-specific standards performed well on their own, we next set out to generate a single vaginal standard to measure bacterial burden in the vaginal micro-environment. We compared standards that utilized common vaginal bacterial micro-organisms and generated standards consisting of different combinations of the G, P, F, and L 16S rRNA genes. This included a standard consisting of all four taxa in equal proportions (G:P:F:L), the BVAB (G:P:F), a standard representing an intermediate community (G:P:L), and an optimal community favoring lactobacilli (1G:9L).

**Figure 2:**
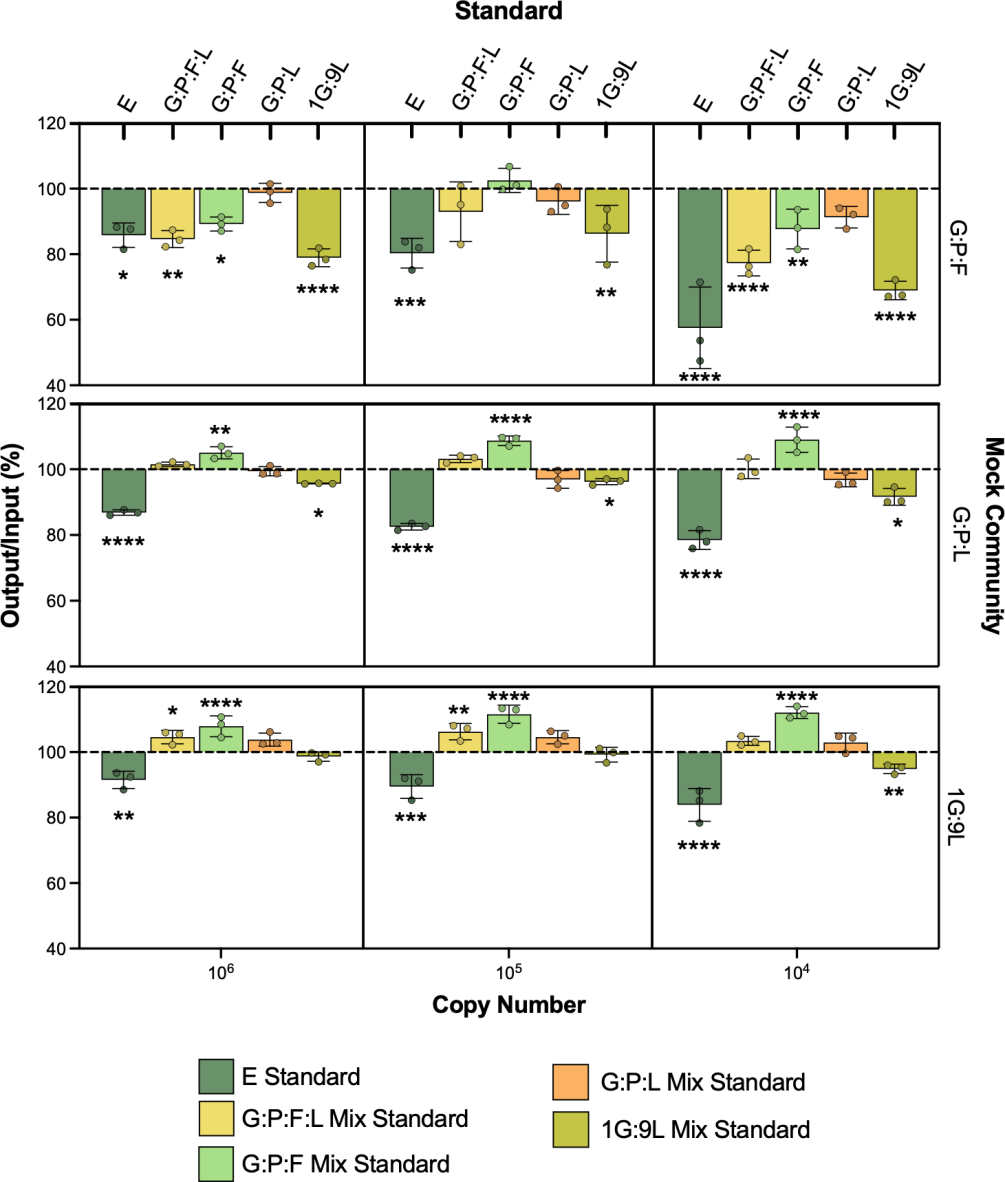
Mock vaginal communities using E and G:P:L mix standard. The equal parts G:P:L community is meant to represent a BV-like vaginal microbiome (top), G:P:L is representative of an intermediate microbiome (middle), and the 1G:9L is meant to represent an optimal vaginal microbiome (bottom). Known copy numbers are used as input, listed on the x-axis. The output is calculated using the corresponding standard and divided by the input to generate a percentage. A 100% output/input indicates the same copy number that was input was read as output from the standard. The resulting output/input was averaged and compared to 100% using two-way ANOVA, *P ≤ 0.05, **P ≤ 0.01, ***P ≤ 0.001, ****P ≤ 0.0001.

Comparing these standards to each other and to the traditional E reference standard, we observed that vaginal mix standards and E standards were similar in efficiency (**Table 1**; **Figures S3A–E**). These standards were then compared using mock vaginal communities, where an optimal vaginal microbiota was represented by a mix of nine parts L to one-part G 16S rRNA gene plasmids, an intermediate community was represented by a mix of equal parts G, P, and L, and a BV-like microbiota consisting of G, P, F (**Figure 2**). Interestingly, the E standard significantly underestimated the mock vaginal communities, indicated by the measured output being less than the known input (all p < 0.05 when compared to 100%). Differences in other mixed vaginal standards in mock communities were observed. The measured output of the G:P:L mixed standard did not significantly differ from the known input in any of the three communities (all p > 0.05 when compared to 100%). Interestingly, this mixed vaginal standard never significantly differed regardless of the copy number of the vaginal community being measured. Whereas, in the E standard, the difference between measurements was greater at decreasing dilutions and was significant when comparing 10^6^ to 10^4^ copy numbers (**Figure S4A**), indicating that the E standard error varied based on the copy number of the 16S rRNA gene in the sample. Overall, these results indicate that the G:P:L mix standard was the most accurate for measuring all tested mock vaginal communities as it did not significantly differ in any dilution.

**Figure 3:**
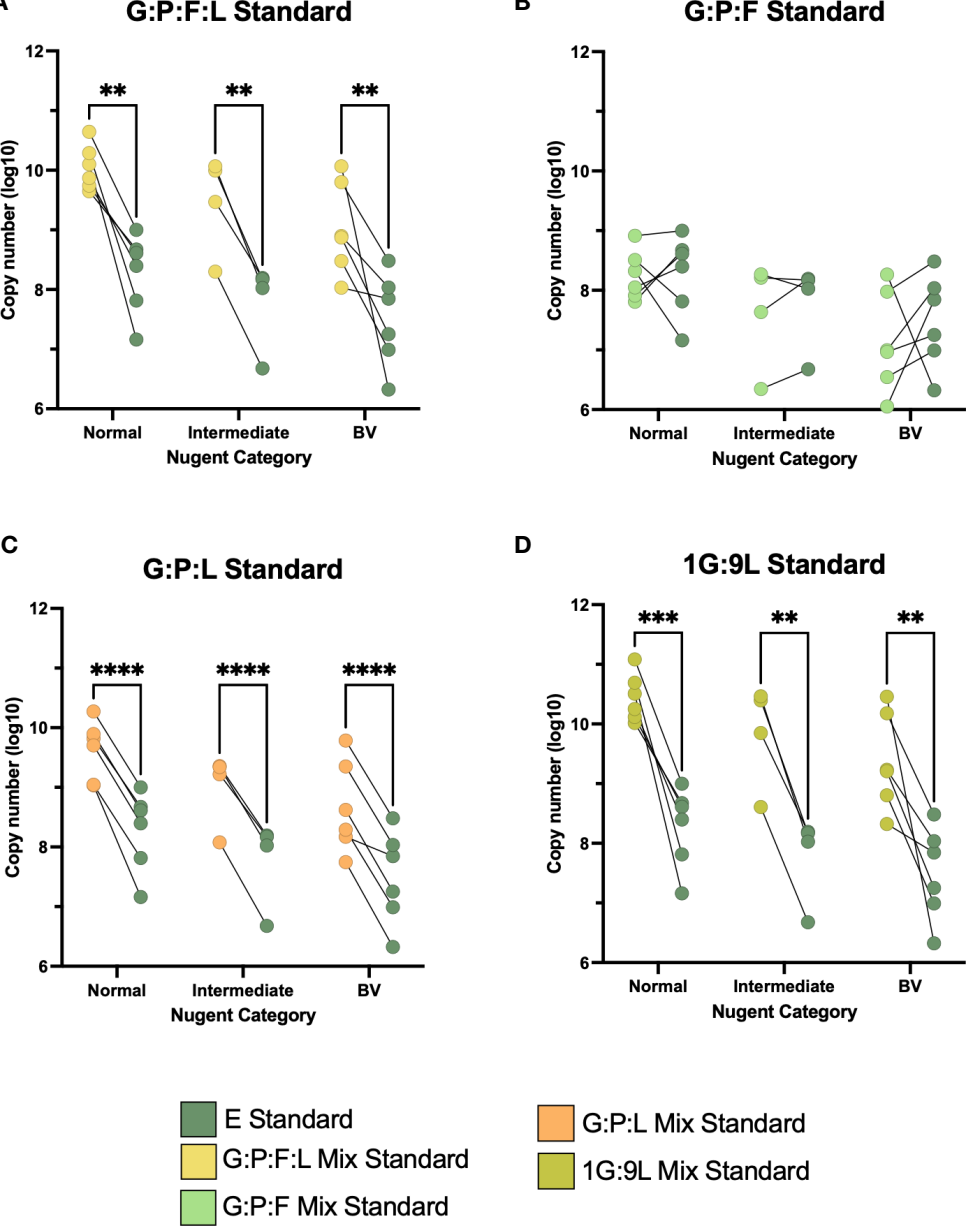
**(A–D)** Paired comparisons between the E and the four vaginal mixed standards across the three categories of Nugent score, normal, intermediate, and BV. The line between two points represents the same vaginal sample with each standard. The variability between these points was compared. All experiments were performed in triplicate and comparisons were made using two-way ANOVA, **P ≤ 0.01, ***P ≤ 0.001, ****P ≤ 0.0001.

We continued to assess these mixed vaginal standards using vaginal samples collected from a diverse group of women enrolled in a previous study (Mott et al., 2021). First, we wanted to determine if this variation was based on the communities within these samples. 16 vaginal samples were stratified based on Nugent score category (6 normal, 4 intermediate, and 6 BV) (Nugent et al., 1991) We measured each sample with the different mixed standards and compared them to the E standard (**Figures 3A–D**). Interestingly, the difference between the G:P:F:L, G:P:L, and 1G:9L standards to the E standard significantly varied regardless of the Nugent category. Samples in all three Nugent categories varied consistently between the vaginal mix standard and the E standard. The G:P:L standard was the most significantly different (all p < 0.0001), followed by 1G:9L (all p < 0.003), and G:P:L:F (all p < 0.01). However, the G:P:F standard was the most similar to the E standard and was not significantly different at any Nugent score category. To determine if there was a variation in copy number similar to that observed in the mock vaginal communities, we compared the difference in the 16S rRNA gene copy number at three different dilutions (**Figure S4B**). The difference between the E and vaginal mix standards became significantly greater at increasing dilutions, supporting the results from the mock vaginal communities, indicating a similar variation based on dilution.

## 4 Discussion

In this study, we generated novel qPCR standards for measuring the bacterial burden in the vaginal micro-environment. These standards were composed of 16S rRNA genes of common vaginal micro-organisms, *Gardnerella* spp., *Prevotella* spp., *Fannyhessea* spp., and *Lactobacillus* spp., and were compared to the traditional E standard. These new standards, specifically the one composed of G:P:L, were able to improve the accuracy of bacterial burden measurements within the vaginal microbial community and the complex BV biofilm.

Strengths of this study include the use of standards that are representative of bacteria that are common members of the vaginal microbiota (Salinas et al., 2020; Pacha-Herrera et al., 2022), rather than *E. coli*, which is not typically present in this micro-environment (Chen et al., 2021). An optimal vaginal microbiota is dominated by *Lactobacillus* spp. such as *L. crispatus, L. jensenii*, and *L. gasseri*, whereas in BV there is an increasing abundance of facultative and strict anaerobic bacteria including *G. vaginalis*, *P. bivia*, and *F. vaginae.* To generate taxa-specific primers, our SP amplicons varied in length. However, we observed that the efficiency between the UP and SP for a given micro-organism did not vary more than 1%. This suggests that the UP region contributes more to the efficiency of the standard rather than to the variation in the length of the SP region.

There are other BVAB present in the vaginal micro-environment and the BV biofilm; therefore, the absolute bacterial burden is difficult to quantify. To help account for this in our study, we created mock vaginal communities where the quantities of 16S rRNA gene copies are known. Since this is only a model of *in vivo* conditions, variables such as biofilm resistance and efficiency of DNA isolation cannot be actively replicated in these mock communities and require additional experimentation and validation (Lima et al., 2022). We tested our mock communities at varying copy numbers, from 10^6^ to 10^4^. This was designed to assess various points along the standard which ranges from 10^7^ to 10^2^ 16S rRNA gene copy numbers. Interestingly, the different combinations of mixed vaginal bacteria performed variably across the different mock communities, and the G:P:L mix standard was observed to be the most accurate as it did not differ from 100% in any case. Perhaps a mixed standard that closely represents an intermediate vaginal community allows for a more robust measurement of bacterial burden across the spectrum of BV. We also observed Nugent-independent differences between the G:P:L and the E standard in a diverse range of vaginal samples.

A limitation of this study is that we chose to generate our mixed vaginal standard using a subset of bacteria found in the vaginal microbiome (Zozaya-Hinchliffe et al., 2010; Gajer et al., 2012). These organisms are most common in both optimal (*Lactobacillus* spp.) and BV (*Gardnerella* spp., *Prevotella* spp., and *Fannyhessea* spp.) vaginal microbiomes. Also, these specific micro-organisms have been found to change in relative abundance in the days leading up to incident BV (Muzny et al., 2018). Additional vaginal bacteria can be tested and combined in standards if there is particular interest moving forward. For example, *Sneathia* spp. can be included. There is growing evidence that *Sneathia* spp. are involved in preterm birth and vaginal inflammation (Anahtar et al., 2015; Fettweis et al., 2019; Łaniewski and Herbst-Kralovetz, 2021) and may be a secondary colonizer to the BV biofilm. Another limitation is that we only tested the V6 region of the 16S rRNA gene because we based our approach on previous work in this area (Bacchetti De Gregoris et al., 2011). We are unsure how standards constructed from other regions would compare to our findings, as primers for different variable regions amplify specific micro-organisms at varying efficiencies (Van Der Pol et al., 2019). The V6 region is similar between species within the same genus, for example different *Lactobacillus* spp. share the same 16S rRNA gene sequence. Thus, we were not able to develop species-specific standards for our micro-organisms of interest, *Gardnerella* spp., *Prevotella* spp., *Fannyhessea* spp., and *Lactobacillus* spp. (Janda and Abbott, 2007; Větrovský and Baldrian, 2013). However, since there are multiple different species of the same genus known to be found in the vaginal microbiota (e.g., *L. crispatus* and *L. gasseri*), genus-specific standards may perform better than ones generated using specific species. A future step would be to test other taxa as well as multiple variable regions of the 16S rRNA gene to identify a standard most optimal for the vaginal microbiota.

Overall, the ability to accurately measure the bacterial burden of the vaginal microbiota will improve our understanding of BV pathogenesis. For example, different Gardnerella species may be present in women who do not have BV, such as *G. vaginalis, G. leopoldii, G. piotii*, and *G. swidsinskii* (Castro et al., 2020), but, perhaps at a certain threshold or level of virulence, *Gardnerella* spp. can overcome the opposing forces in the vaginal micro-environment to initiate BV biofilm development (Roy et al., 1994). A better understanding of this sequence of events and changes in bacterial burden over time will help inform improvements in BV diagnosis and treatment. This method can also be synergized with complementary bioinformatics approaches such as calculating inferred absolute abundance (Tettamanti Boshier et al., 2020). In this method, the total bacterial burden is multiplied by the relative abundance through 16S rRNA gene sequencing to obtain a value comparable to the burden of the specific vaginal bacterial species. Improving the method to calculate the bacterial burden will result in improved overall accuracy of this calculation. Accurate bacterial burden measurements will also improve vaginal microbiome studies where vaginal sample collection takes place. Since this is a relatively cost-effective method, bacterial burden can be determined to guide selection and prioritization of samples for deeper sequencing. This method can also be adopted to better understand the etiology of incident BV. Dramatic bacterial shifts can occur in the vaginal micro-environment within short periods of time (Gajer et al., 2012), and high resolution of these changes is required to elucidate bacterial dynamics in the pathogenesis of BV. We plan to use our newly generated standard in longitudinal studies in combination with inferred absolute abundance to enhance our understanding of the changes in the burden of key vaginal bacteria prior to the onset of incident BV.

## Data availability statement

The original contributions presented in the study are included in the article/**Supplementary Material**. Further inquiries can be directed to the corresponding author.

## Ethics statement

The studies involving human participants were reviewed and approved by Louisiana State University Health Sciences Center [LSUHSC] IRB protocol #1081. The patients/participants provided their written informed consent to participate in this study.

## Author contributions

JE and CA conceived this study. JE performed the experiments. JE and CA analyzed the data. RL clinically categorized patient samples and established the sample bank. NC, ET, PL, MH-K, AQ, CM, CT jointly supervised the study and provided oversight during the manuscript editing process All authors contributed to the article and approved the submitted version.

## Supplementary material

Supplementary Figure 1
**(A)** qPCR diagram. **(B)** pGEM-T Easy vector diagram with 16S rRNA gene.

Supplementary Figure 2 Relative abundance heatmap for vaginal samples used in this study.

Supplementary Figure 3
**(A–E)** qPCR standards using UP for *Escherichia coli* (E) and vaginal mixed standards composed of *Gardnerella* spp. (G): *Prevotella* spp. (P): *Fannyhessea* spp. (F), and *Lactobacillus* spp. (L). Data points are generated from three-fold dilutions of the purified plasmids starting at 10^7^ copies to 10^2^ copies per reaction. Each point represents an average of 3 replicate qPCR reactions with standard deviation error bars. Efficiency (E) is calculated based on the slope of the linear regression. Axes are labeled by the quantification cycle (Cq) and the number of copies per reaction (log10).

Supplementary Figure 4
**(A)** E standard for the 3 mock communities showing a decrease in accuracy. **(B)** The E and G:P:L mix standard were performed on DNA isolated from vaginal swabs. These standards were performed at three ten-fold dilutions D1 (1/10), D2 (1/100), and D3 (1/1000). The difference in the resulting copy number from the E and G:P:L mix standard was calculated and compared across the three dilutions.

Supplementary Table 1
**(A)** qPCR information. **(B)** Metadata for patients. **(C)** Statistical tests.
