# Supplementary material for: A novel Gardnerella, Prevotella, and Lactobacillus standard that improves accuracy in quantifying bacterial burden in vaginal microbial communities
Source: Front Cell Infect Microbiol. 2023 Jun 19;13:1198113. doi: 10.3389/fcimb.2023.1198113 (PMC10315654; doi:10.3389/fcimb.2023.1198113)

Supplemental Figure 1

A

Two-step qPCR protocol

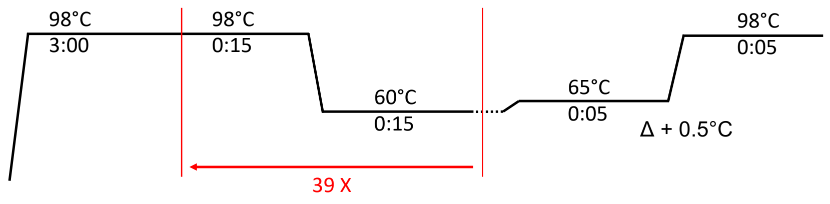

B

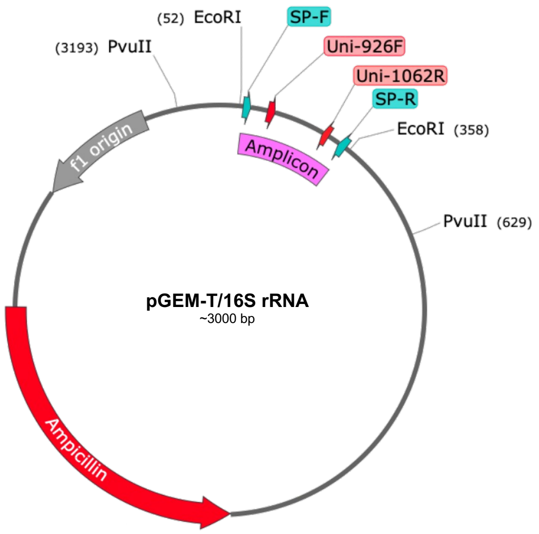

Supplemental Figure 2

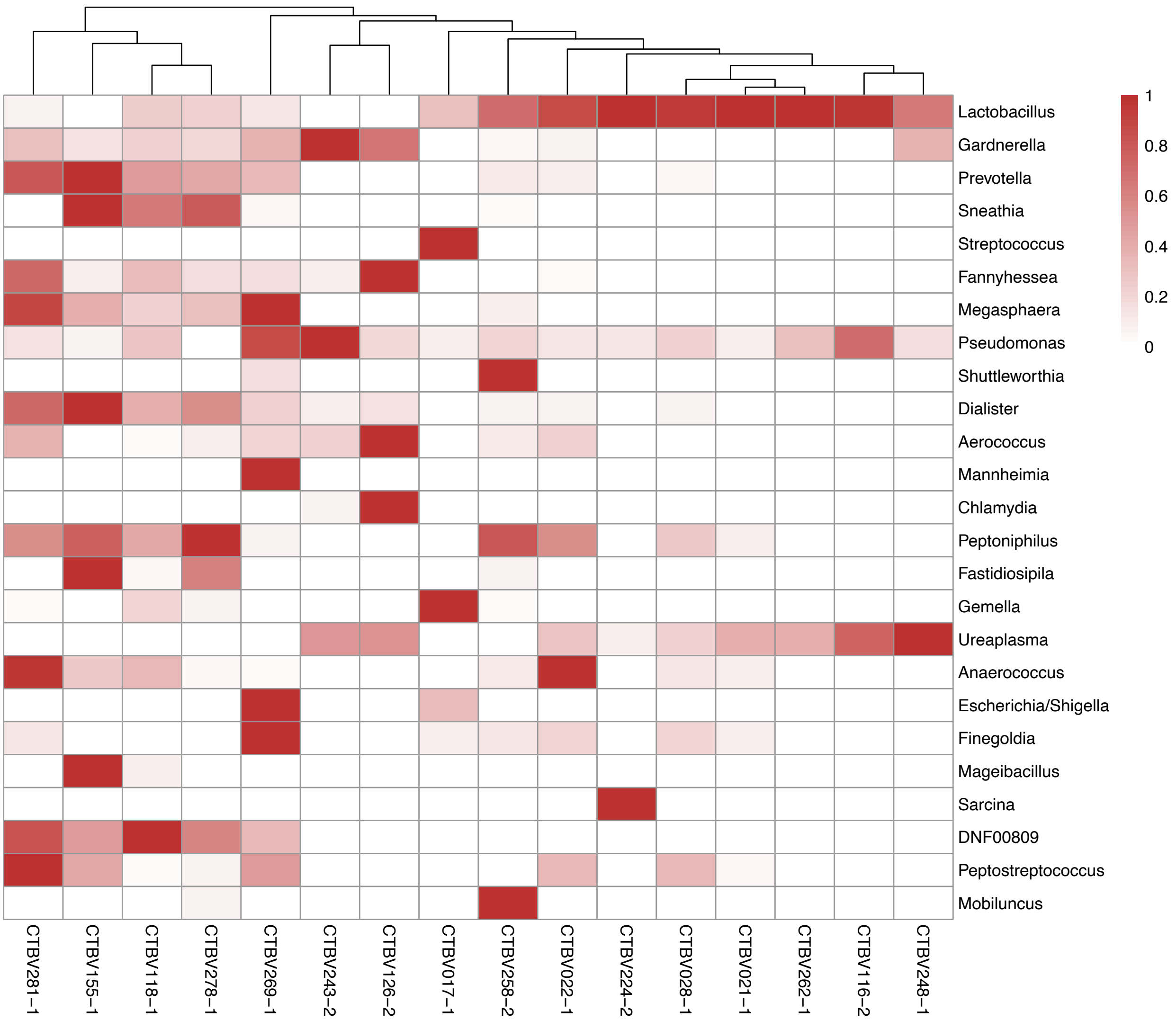

Supplemental Figure 3

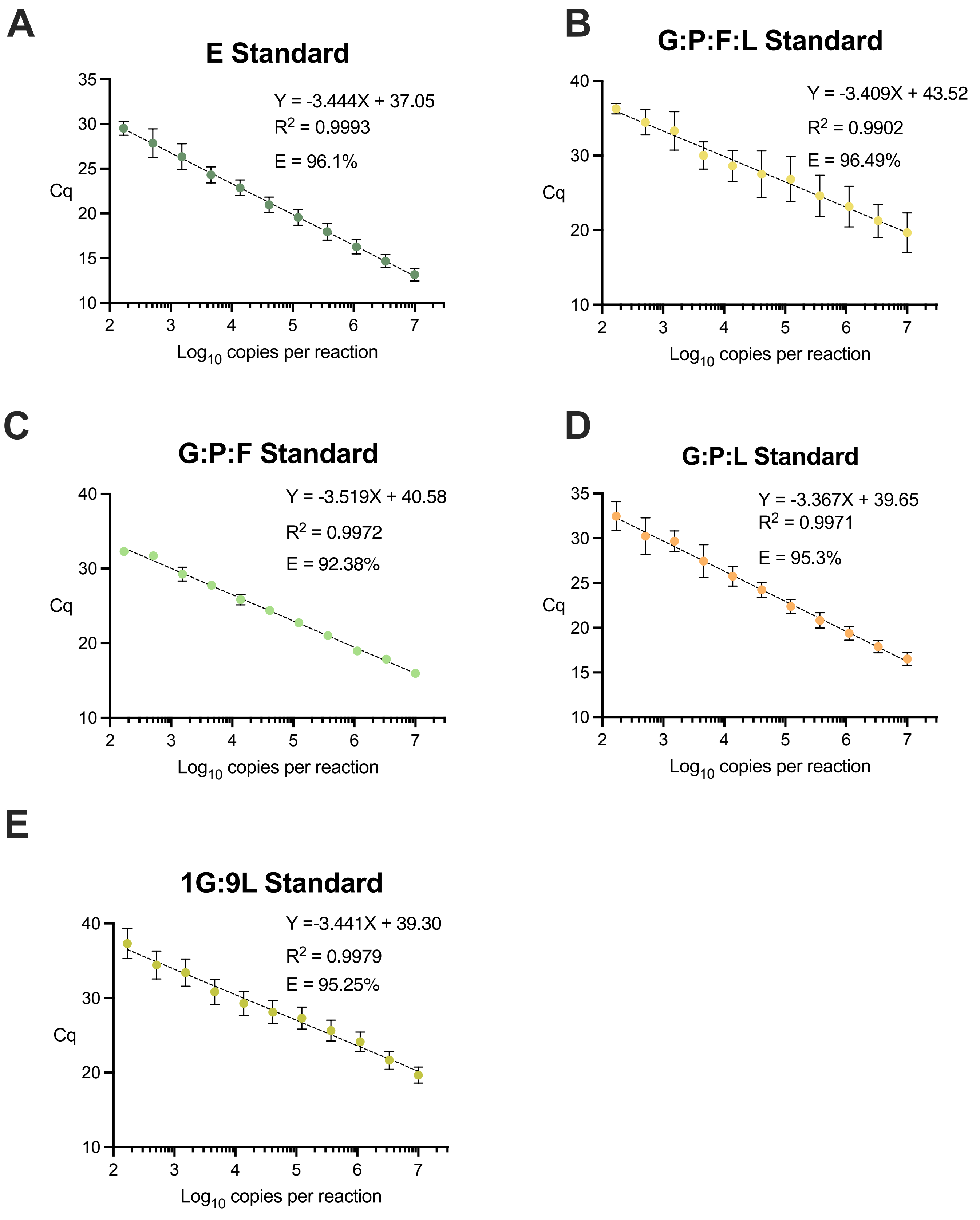

Supplemental Figure 4

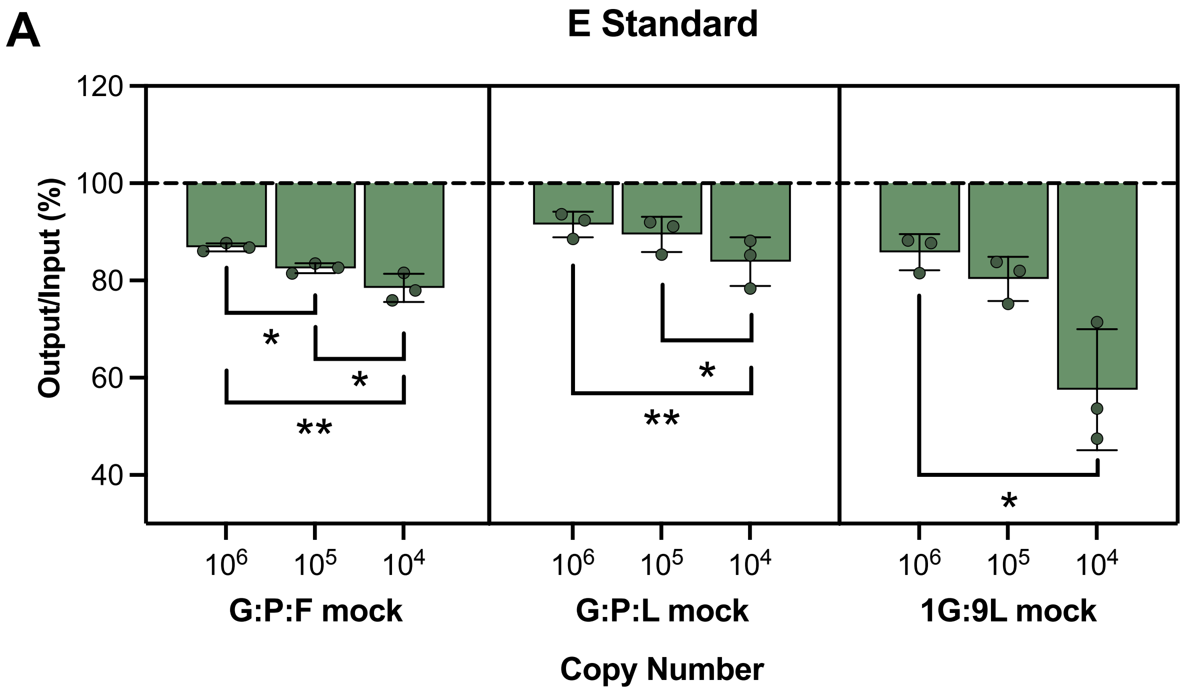

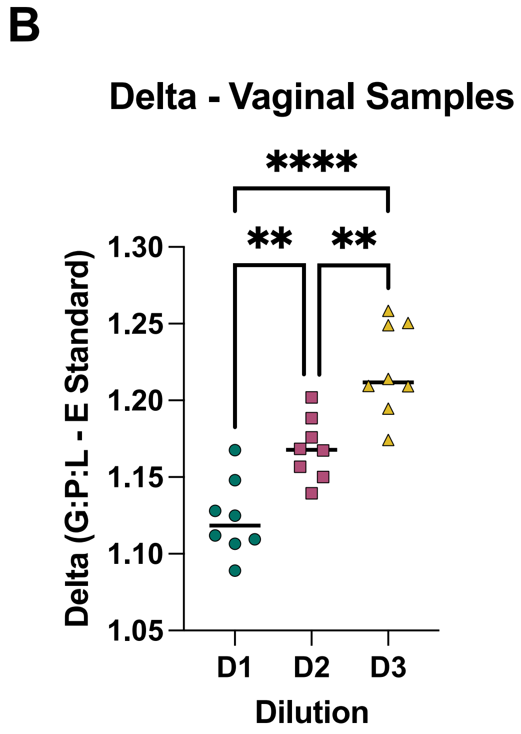

Supplement: Supplementary Figure 1 — (A) qPCR diagram. (B) pGEM-T Easy vector diagram with 16S rRNA gene. [file DataSheet_1.pdf]
